# Hybrid fNIRS-EEG based classification of auditory and visual perception processes

**DOI:** 10.3389/fnins.2014.00373

**Published:** 2014-11-18

**Authors:** Felix Putze, Sebastian Hesslinger, Chun-Yu Tse, YunYing Huang, Christian Herff, Cuntai Guan, Tanja Schultz

**Affiliations:** ^1^Cognitive Systems Lab, Institute of Anthropomatics and Robotics, Karlsruhe Institute of TechnologyKarlsruhe, Germany; ^2^Department of Psychology, Center for Cognition and Brain Studies, The Chinese University of Hong KongHong Kong, China; ^3^Temasek Laboratories, National University of SingaporeSingapore, Singapore; ^4^Nuffield Department of Clinical Neurosciences, John Radcliffe HospitalOxford, UK; ^5^Institute for Infocomm Research (I2R), A^*^STARSingapore, Singapore

**Keywords:** brain-computer interface, EEG, fNIRS, visual and auditory perception

## Abstract

For multimodal Human-Computer Interaction (HCI), it is very useful to identify the modalities on which the user is currently processing information. This would enable a system to select complementary output modalities to reduce the user's workload. In this paper, we develop a hybrid Brain-Computer Interface (BCI) which uses Electroencephalography (EEG) and functional Near Infrared Spectroscopy (fNIRS) to discriminate and detect visual and auditory stimulus processing. We describe the experimental setup we used for collection of our data corpus with 12 subjects. On this data, we performed cross-validation evaluation, of which we report accuracy for different classification conditions. The results show that the subject-dependent systems achieved a classification accuracy of 97.8% for discriminating visual and auditory perception processes from each other and a classification accuracy of up to 94.8% for detecting modality-specific processes independently of other cognitive activity. The same classification conditions could also be discriminated in a subject-independent fashion with accuracy of up to 94.6 and 86.7%, respectively. We also look at the contributions of the two signal types and show that the fusion of classifiers using different features significantly increases accuracy.

## 1. Introduction

For the last decade, multimodal user interfaces have become omnipresent in the field of human-computer interaction and in commercially available devices (Turk, 2014). Multimodality refers to the possibility to operate a system using multiple input modalities but also to the ability of a system to present information using different output modalities. For example, a system may present information on a screen using text, images and videos or it may present the same information acoustically by using speech synthesis and sounds. However, such a system has to select an output modality for each given situation. One important aspect it should consider when making this decision is the user's workload level which can negatively influence task performance and user satisfaction, if too high. The output modality of the system which imposes the smaller workload on the user does not only depend on the actions of the system itself, but also on concurrently executed cognitive tasks. Especially in dynamic and mobile application scenarios, users of a system are frequently exposed to external stimuli from other devices, people or their general environment.

According to the multiple resource theory of Wickens (2008), the impact of a dual task on the workload level depends on the type of cognitive resources which are required by both tasks. If the overlap is large, the limited resources have to be shared between both tasks and overall workload will increase compared to a pair of tasks with less overlap, even if the total individual task load is identical. For example, Yang et al. (2012) showed a study in which they combine a primary driving task with additional auditory and visual task of three difficulty levels. They showed that the difference in the performance level of the driving task depends on the modality of the secondary task: According to their results, secondary visual tasks had a stronger impact on the driving than secondary auditory tasks, even if individual workload of the auditory tasks was slightly higher than of the visual tasks. For Human-Computer Interaction (HCI), this implies that when the interaction strategy of the system must must select from different output channels by which it can transfer information to the user, its behavior should take into account the cognitive processes which are already ongoing. It is possible to model the resource demands of cognitive tasks induced by the system itself (see for example Cao et al., 2009). For example, we know that presenting information using speech synthesis requires auditory perceptual resources while presenting information using a graphical display will require visual perceptual resources. However, doing the same for independent parallel tasks is impossible in an open-world scenario where the number of potential distractions is virtually unlimited. Therefore, we have to employ sensors to infer which cognitive resources are occupied.

To some degree, perceptual load can be estimated from context information gathered using sensors like microphones or cameras. However, if, for example, the user wears earmuffs or head phones, acoustic sensors cannot reliably relate acoustic scene events to processes of auditory perception. Therefore, we need a more direct method to estimate those mental states. A Brain-Computer Interface (BCI) is a “system that measures central activity and converts it into artificial output that replaces, restores, enhances supplements, or improves natural central nervous system output” (Wolpaw and Wolpaw, 2012). BCIs can therefore help to detect or discriminate perceptual processes for different modalities directly from measures of brain activity and are therefore strong candidates to reliably discriminate and detect modality-specific perceptual processes. As BCIs have many additional uses for active interface control or for passive user monitoring, they may be already in place for other tasks and would not require any additional equipment.

Our system combines two different signal types [Electroencephalography (EEG) and functional Near Infrared Spectroscopy (fNIRS)] to exploit their complementary nature and to investigate their individual potential for classifying modality-specific perceptual processes: EEG is the traditional signal for BCIs, recording electrical cortical activity using electrodes. fNIRS on the other hand captures the hemodynamic response by exploiting the fact that oxygenated and de-oxygenated blood absorb different proportions of light of different wavelengths in the near-infrared spectrum. fNIRS captures different correlates of brain activity than EEG: While EEG measures an electrical process, fNIRS measures metabolic response to cognitive activity. This fact makes it plausible that a fusion of both signal types can give a more robust estimation of a person's cognitive state.

BCIs based on EEG have been actively researched since the 1970s, for example in computer control for locked-in patients (e.g., Wolpaw et al., 1991; Sitaram et al., 2007). BCIs based on fNIRS have become increasingly popular since the middle of last decade (Sitaram et al., 2007). The term hybrid BCI generally describes a combination of several individual BCI systems (or the combination of a BCI with another interface) (Pfurtscheller et al., 2010). A sequential hybrid BCI employs two BCIs one after another. One application of a sequential BCI is to have the first system act as a “brain switch” to trigger the second system. A sequential hybrid BCI usually resorts to different types of brain activity measured by a single signal type (e.g., correcting mistakes of a P300 speller by detecting error potentials, Spüler et al., 2012). In contrast, a simultaneous hybrid BCI system usually combines entirely different types of brain signals to improve the robustness of the joint system. The first simultaneous hybrid BCI that is based on synchronous measures of fNIRS and EEG was proposed by Fazli et al. (2012) for classification of motor imagery and motor execution recordings. The authors reported an improvement in recognition accuracy by combining both signal types.

Zander and Kothe (2011) defined Passive BCI as follows: “a passive BCI is one that derives its outputs from arbitrary brain activity arising without the purpose of voluntary control, for enriching a humanmachine interaction with implicit information on the actual user state.” A number of such systems exist to classify the user's workload level, for example presented by Heger et al. (2010) or Kothe and Makeig (2011). Those systems used different EEG feature extraction techniques that are usually related to the frequency power distribution to classify low and high workload conditions. Other researchers derived features from Event Related Potentials (ERPs) in time domain (Allison and Polich, 2008; Brouwer et al., 2012) or used Common Spatial Patterns (Dijksterhuis et al., 2013) to discriminate workload levels. Workload level is typically assessed from subjective questionnaires or task difficulty. Sassaroli et al. (2008) placed fNIRS optodes on the forehead to measure concentration changes of oxyhemoglobin and deoxyhemoglobin in the prefrontal cortex during memory tasks and discriminated between three different levels of workload in three subjects. Similarly, Bunce et al. (2011) discriminate different workload levels for a complex Warship Commander Task, for which task difficulty was manipulated to create different levels of workload. They recorded fNIRS from 16 optodes at the dorsolateral prefrontal cortex and saw significant differences in oxygenation between low and high workload conditions. They also observed a difference in signal response to different difficulty settings for expert and novice users, which was mirrored by the behavioral data. Herff et al. (2014) showed that it is possible to classify different levels of n-back difficulty corresponding to different levels of mental workload on a single trials for prefrontal fNIRS signals with an accuracy of up to 78%. Hirshfield et al. (2009) combined EEG and fNIRS data for workload estimation in a counting task and saw better results for fNIRS in comparison to frequency based EEG-features. The authors reported surprisingly low accuracy for their EEG-based classifier and suspected problems with coverage of relevant sites and montage-specific artifacts. In contrast, Coffey et al. (2012) presented results from a similar study but showed worse results for the fNIRS features. From the available literature, it is hard to judge the relative discriminative power of the different signal types. On the one hand, Coffey et al. (2012) and Hirshfield et al. (2009) cover only a small aspect of general passive BCI research as they both concentrate on the classification of workload and use similar fNIRS montages. On the other hand, the experiments are too different to expect identical results (different cognitive tasks, different features, etc.). Therefore, there is too little data available for a final call on the synergistic potential between both modalities and their applicability to specific classification tasks. This paper contributes to an answer of this question by investigating a very different fNIRS montage, by including different types of EEG features to ensure adequate classification accuracy and by looking at a more specific aspect of cognitive activity, namely processing of different input modalities.

All the systems mentioned above modeled workload as a monolithic construct and did not classify the resource types which contributed to a given overall workload level. While there exist user studies, e.g., Heger et al. (2011), which show that it is possible to improve human-computer interaction using this construct, many use cases—like the mentioned selection between auditory and visual output modalities—require a more fine grained model of mental workload, like the already mentioned multiple resource theory (Wickens, 2008). Neural evidence from a study by Keitel et al. (2012) of subjects switching between bimodal and unimodal processing also indicated that cognitive resources for visual and auditory processing should be modeled separately. Most basic visual processing takes place in the visual cortex of the human brain, located in the occipital lobe, while auditory stimuli are processed in the auditory cortex located in the temporal lobes. This clear localization of important modality-specific areas in the cortex accessible for non-invasive sensors hints at the feasibility of separating both types of processing modes.

In this paper, we investigate how reliably a hybrid BCI using synchronous EEG and functional fNIRS signals can perform such classification tasks. We describe an experimental setup in which natural visual and auditory stimuli are presented in isolation and in parallel to the subject of which both EEG and fNIRS data is recorded. On a corpus of 12 recorded sessions, we train BCIs using features from one or both signal types to differentiate and detect the different perceptual modalities. This paper contributes a number of substantial findings to the field of passive BCIs for HCI: We trained and evaluated classifiers which can either discriminate between predominantly visual and predominantly auditory perceptual activity or which were able to detect visual and auditory activity independently of each other. The latter is ecologically important as many real-life tasks demand both visual and auditory resources. We showed that both types of classifiers achieved a very high accuracy both in a subject-dependent and subject-independent setup. We investigated the potential of combining different feature types derived from different signals to achieve a more robust and accurate recognition result. Finally, we look at the evaluation of the system on continuous data.

## 2. Materials and methods

### 2.1. Participants

Twelve healthy young adults (6 male, 6 female), age between 21 and 30 years (mean age 23.6, *SD* 2.6 years) without any known history of neurological disorders participated in this study. All of them have normal or corrected-to-normal visual acuity, normal auditory acuity, and were paid for their participation. The experimental protocol was approved by the local ethical committee of National University of Singapore, and performed in accordance with the policy of the Declaration of Helsinki. Written informed consent was obtained from all subjects and the nature of the study was fully explained prior to the start of the study. All subjects had previous experience with BCI operation or EEG/fNIRS recordings.

### 2.2. Experimental procedure

Subjects were seated in a sound-attenuated room with a distance of approximately one meter from a widescreen monitor (24″ BenQ XL2420T LED Monitor, 120 Hz, 1920 × 1080), which was equipped with two loudspeakers on both sides (DELL AX210 Stereo Speaker). During the experiment, subjects were presented with movie and audio clips, i.e., silent movies (no sound; VIS), audiobooks (no video; AUD), and movies with both video and audio (MIX). We have chosen natural, complex stimuli in contrast to more controlled, artificially generated stimuli to keep subjects engaged with the materials and to achieve a realistic setup.

Besides any stimulus material, the screen always showed a fixation cross. Subjects were given the task to look at the cross at all times to avoid an accumulation of artifacts. When there was no video shown, e.g., during audio clips and during rest periods, the screen pictured the fixation cross on a dark gray background. In addition to the auditory, visual and audiovisual trials, there were IDLE trials. During IDLE, we showed a dark gray screen with a fixation cross in the same way as during the rest period between different stimuli. Therefore, subjects were not be able to distinguish this condition from the rest period. In contrast to the rest periods, IDLE trials did not follow immediately after a segment of stimulus processing and can therefore be assumed to be free of fading cognitive activity. IDLE trials were assumed to not contain any systematic processing of stimuli. While subjects received other visual or auditory stimulations from the environment during IDLE trials, those stimulations were not task relevant and of lesser intensity compared to the prepared stimuli. In contrast to AUD, VIS, and MIX trials, there was no additional resting period after IDLE trials.

**Figure 1:**
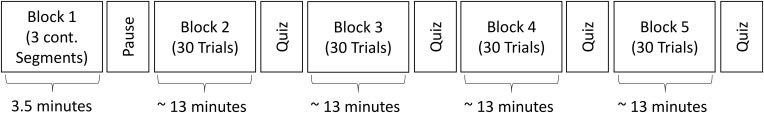
**Block design of the experimental setup**.

**Figure 2:**
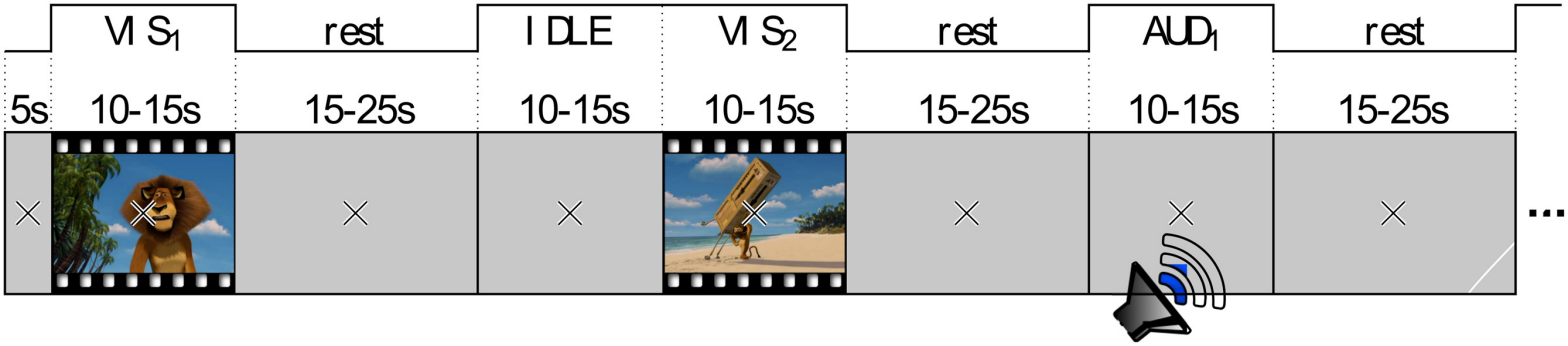
**Example of four consecutive trials with all perceptual modalities**.

The entire recording, which had a total duration of nearly 1 h, consisted of five blocks. Figure 1 gives an overview of the block design. The first block consisted of three continuous clips (60 s audio, 60 s video, 60 s audio and video with a break of 20 s between each of them. This block had a fixed duration of 3 min 40 s. The remaining four blocks had random durations of approximately 13 min each. The blocks 2–5 followed a design with random stimulus durations of 12.5 ± 2.5 s (uniformly distributed) and rest periods of 20 ± 5 s (uniformly distributed). The stimulus order of different modalities was randomized within each block. However, there was no two consecutive stimuli of the same modality. Figure 2 shows an example of four consecutive trials in the experiment. Counted over all blocks, there were 30 trials of each category AUD, VIS, MIX, and IDLE.

The stimuli of one modality in one block formed a coherent story. During the experiment, subjects were instructed to memorize as much of these stories (AUD/VIS/MIX story) as possible. In order to ensure that subjects paid attention to the task, they filled out a set of multiple choice questions (one for each story) after each block. This included questions on contents, e.g., “what happens after…?”, as well as general questions, such as “how many different voices appeared?” or “what was the color of …?”. According to their answers, all subjects paid attention throughout the entire experiment. In the auditory condition, subjects achieved an averaged correct answer rate of 85%, whereas in the visual condition there is a correct answer rate of 82%.

### 2.3. Data acquisition

**Figure 3:**
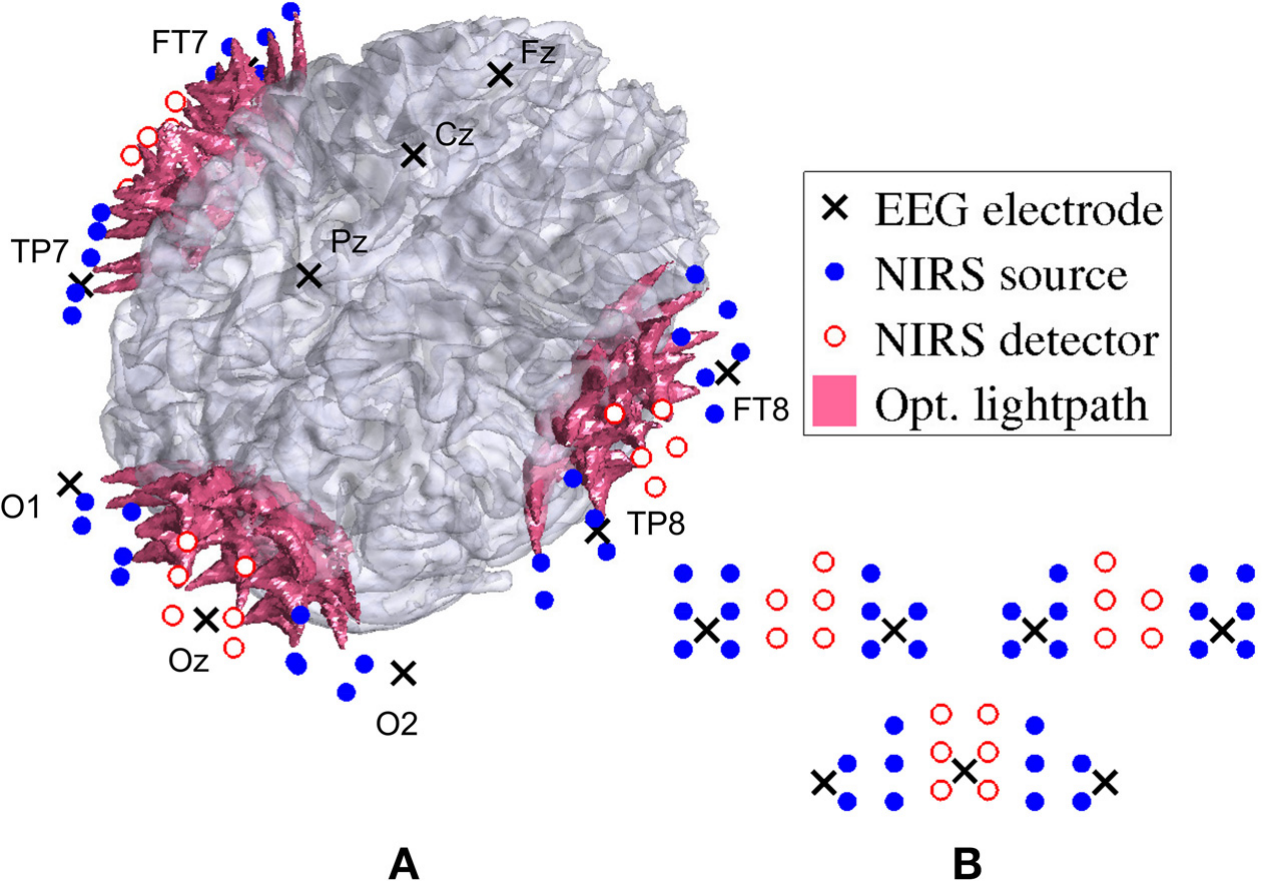
**Locations of EEG electrodes, fNIRS optrodes, and their corresponding optical lightpath**. The arrangement of fNIRS sources and detectors is shown projected on the brain in **(A)** and as unwrapped schematic in **(B)** for the two auditory probes (top left and right) and the visual probe (bottom).

For fNIRS recording, a frequency-domain oximeter (Imagent, ISS, Inc., Champaign, IL, USA) was employed. Frequency-modulated near-infrared light from laser diodes (690 nm or 830 nm, 110 MHz) was conducted to the participants head with 64 optical source fibers (32 for each wavelength), pairwise co-localized in light source bundles. A rigid custom-made head-mount system (montage) was used to hold the source and detector fibers to cover three different areas on the head: one for the visual cortex and one on each side of the temporal cortex. The multi-distance approach as described in Wolf et al. (2003); Joseph et al. (2006) was applied in order to create overlapping light channels. Figure 3 shows the arrangement of sources and detectors in three probes (one at the occipital cortex and two at the temporal lobe). For each probe, two columns of detectors were placed between two rows of sources each to the left and the right, at source-detector distances of 1.7–2.5cm. See Figure 3A for the placement of the probes and Figure 3B for the arrangement of the sources and detectors. After separating source-detector pairs of different probes into three distinct areas, there were a total of 60 channels on the visual probe and 55 channels on each auditory probe. Thus, there was a total number of *n_c_* = 170 channels. The sampling frequency used was 19.5Hz.

EEG was simultaneously recorded with an asalab ANT neuro amplifier and digitized with a sampling rate of 256 Hz. The custom-made head-mount system, used for the optical fibers, also enabled us to place the following 12 Ag/AgCl electrodes according to the standard 10–20 system: Fz, Cz, Pz, Oz, O1, O2, FT7, FT8, TP7, TP8, M1, M2. Both M1, and M2 were used as reference.

After the montage was positioned, the locations of fNIRS optrodes, EEG electrodes, as well as the nasion, pre-auricular points and 123 random scalp coordinates were digitized with Visor (ANT BV) and ASA 4.5 3D digitizer. Using each subject's structural MRI, these digitized points were then coregistered, following Whalen et al. (2008), in order to have all subjects' data in a common space.

### 2.4. Preprocessing

The preprocessing of both fNIRS and EEG data were performed offline. Optical data included an AC, a DC, and a phase component; however, only the AC intensities were used in this study. Data from each AC channel were normalized by dividing it by its mean, pulse-corrected following Gratton and Corballis (1995), median filtered with a filter length of 8 s, and downsampled from 19.5 to 1 Hz. The downsampled optical density changes ΔOD_*c*_ were converted to changes in concentration of oxyhemoglobin (HbO) and deoxyhemoglobin (HbR) using the modified Beer-Lambert law (MBLL) (Sassaroli and Fantini, 2004).

The parameters for differential path-length factor and wavelength-dependent extinction coefficient within this study were based on standard parameters in the HOMER2 package, which was used for conversion process (Huppert et al., 2009). Values of molar extinction coefficients were taken from http://omlc.ogi.edu/spectra/hemoglobin/summary.html^1^. Finally, common average referencing (CAR) was applied to the converted data in order to reduce noise and artifacts that are common in all channels (Ang et al., 2012). Thereby, the mean of all channels is substracted from each individual channel *c*. It is performed on both ΔHbO and ΔHbR.

EEG data were preprocessed with EEGLAB 2013a (Delorme and Makeig, 2004). First the data was bandpass filtered in the range of 0.5–48 Hz using a FIR filter of standard filter order of 6 (= $\frac{3}{\text{low cutoff}}$ · sampling rate). Then, non-brain artifacts were rejected using Independent Component Analysis (ICA) as proposed by Jung et al. (2000). In this process, all 10 channels were converted to 10 independent components. One component of each subject was rejected based on prefrontal eye blink artifacts. Finally, the prestimulus mean of 100 ms was substracted from all stimulus-locked data epochs.

### 2.5. Grand averages

In the following, we calculate Grand Averages of both fNIRS and EEG signals (in time domain and frequency domain) for the different types of stimuli. This is done to investigate the general sensitivity of the signals to differences in modality and to motivate the feasibility of different feature types which we define later for classification.

**Figure 4:**
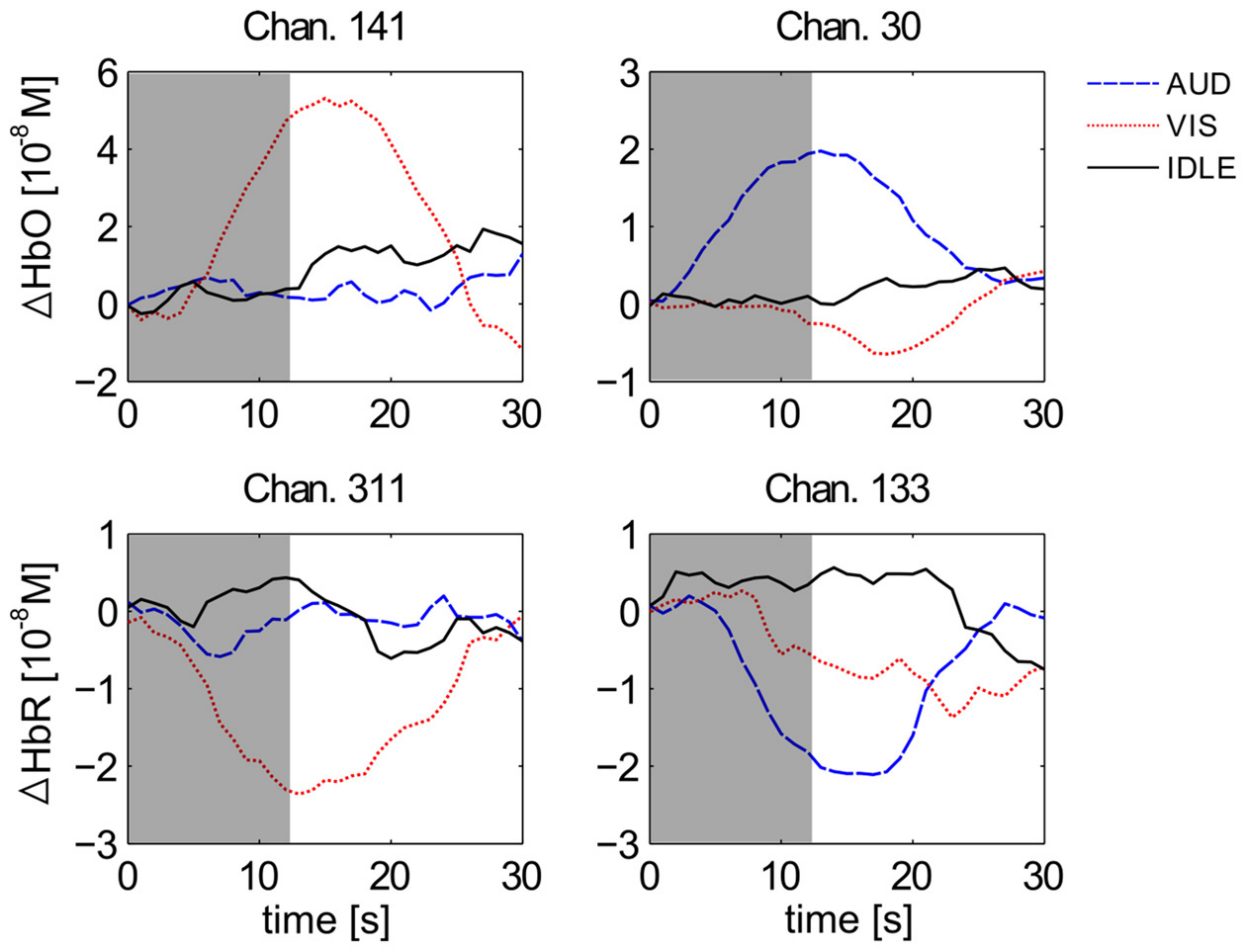
**Grand averaged HRFs of HbO (top) and HbR (bottom) for visual (left) and auditory (right) channels**. Depicted are averages for the classes AUD (blue), VIS (red), and IDLE (black). The area shaded in gray marks the average duration of a stimulus presentation.

Figure 4 shows the averaged haemodynamic response function (HRF) for selected channels of all 12 subjects for labels AUD (blue), VIS (red), and IDLE (black). The stimulus locked data trials (blocks 2–5) are epoched by extracting the first 10 s of each stimulus, and a 2 s prestimulus baseline was substracted from each channel. There was a clear peak in the HRF in response to a VIS stimulus on channels from the occipital cortex (channels 141 and 311 in the figure) and a return to baseline after the stimulus is over after 12.5 s. This effect is absent for an AUD stimulus. Conversely, the channels from the auditory cortex (channels 30 and 133 in the figure) react much stronger to a AUD than to a VIS stimulus.

**Figure 5:**
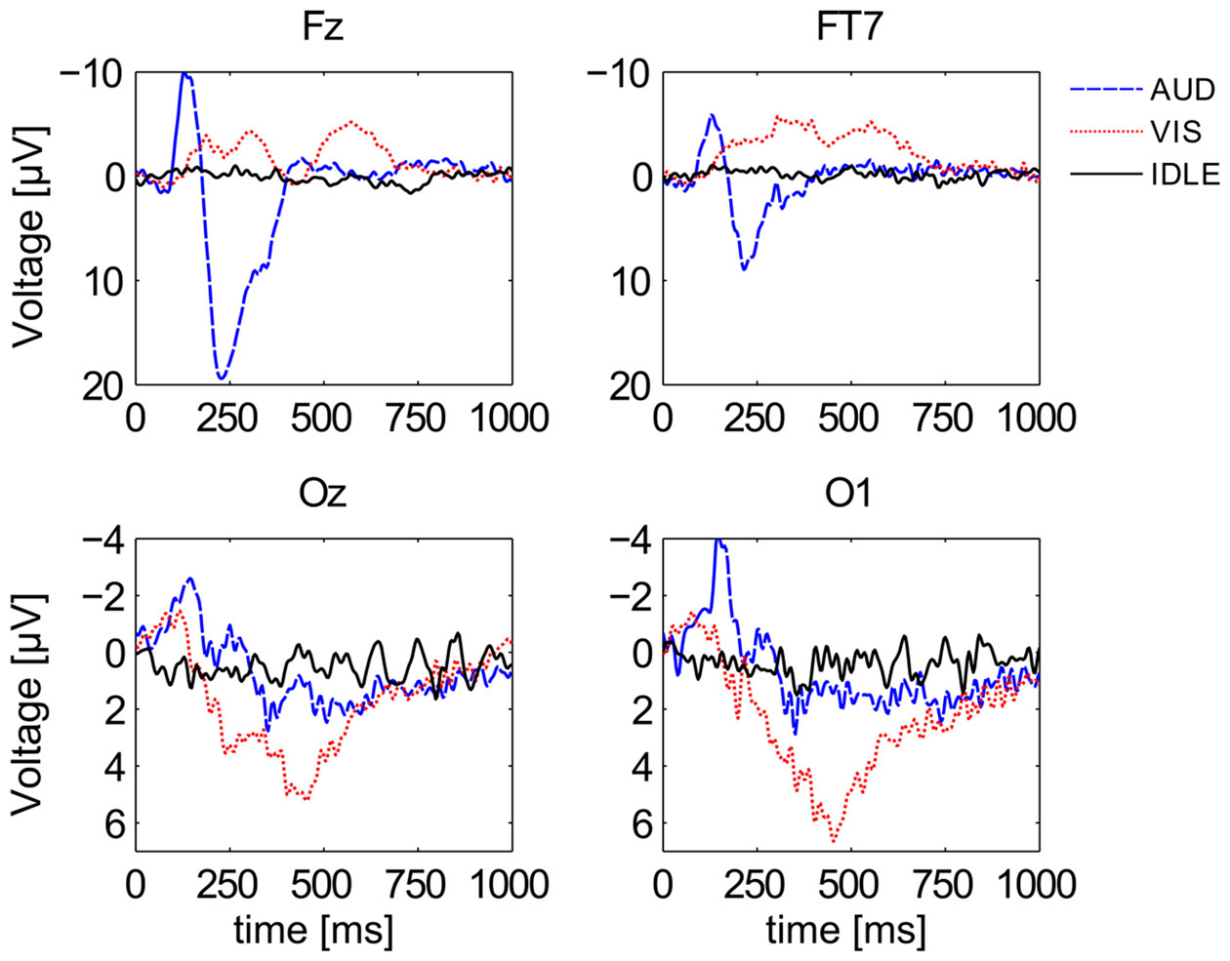
**Grand averaged ERPs of all 3 conditions at 4 different channel locations**. Depicted are averages for the classes AUD (blue), VIS (red), and IDLE (black).

**Figure 6:**
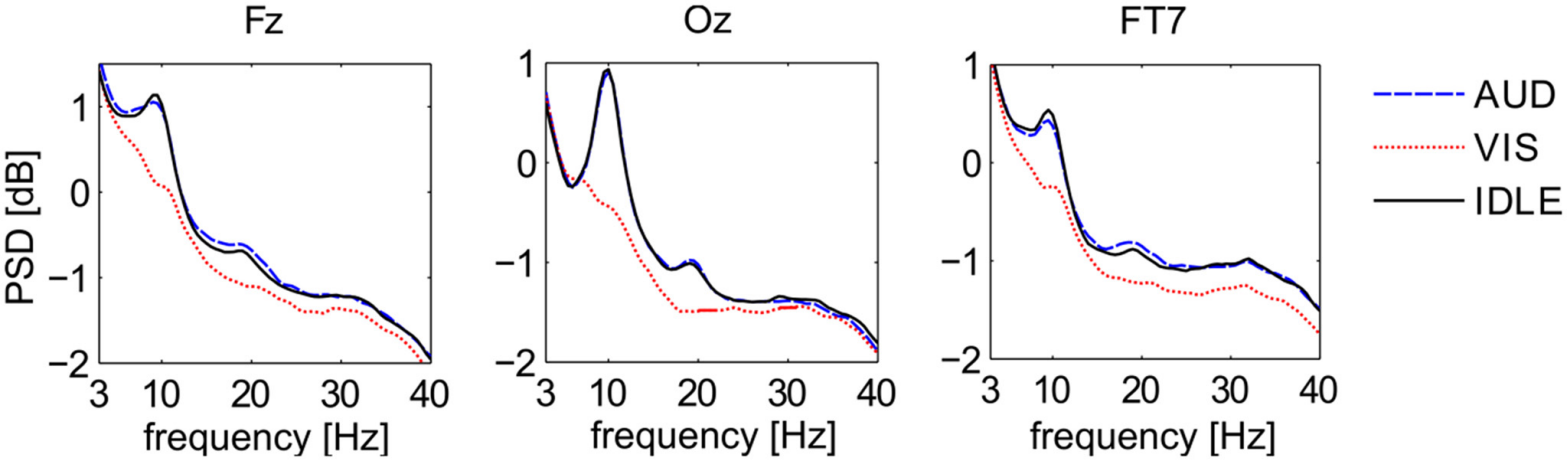
**Power Spectral Density of three EEG signals at Fz, Oz, FT7 for three different conditions**. Depicted are averages for the classes AUD (blue), VIS (red), and IDLE (black).

Figure 5 shows the first second of ERP waveforms of conditions AUD (blue), VIS (red), and IDLE (black), averaged across all 12 subjects. It shows distinctive pattern for auditory and visual stimuli when comparing electrodes at the visual cortex with electrodes at more frontal positions. It is also widely known that frequency responses can be used to identify cognitive processes. Figure 6 shows power spectral density on a logarithmic scale at a frontal midline position (Fz), at the ocipital cortex (Oz) and the temporal lobe (FT7). The plots indicate that especially visual activity can be easily discriminated from auditory activity an no perceptual activity. This fact becomes especially evident at electrode site Oz. The alpha peak for the AUD condition is expected, but unusually pronounced. We attribute this to the fact that the VIS stimuli are richer compared to the AUD stimuli as they often contain multiple parallel points of interest and visual attractors at once. The difference between VIS and AUD trials does also not only involve perceptual processes but also other aspects of cognition, as they differ in content, processing codes and other parameters. On the one hand, this is a situation specific to the scenario we employed. On the other hand, we argue that this difference between visual and auditory information processing pertains for most natural conditions. We will investigate this issue by looking at the discriminability of AUD and IDLE conditions and also at the influence of alpha power on overall performance.

### 2.6. Classification

In this study, we first aimed to classify auditory against visual perception processes. Second, we wanted to detect auditory or visual processes, i.e., we classify modality-specific activity vs. no activity. Third, we wanted to detect a certain perception process in presence of other perception processes.

To demonstrate the expected benefits of combining the fNIRS and EEG signals, we first explored two individual classifiers for each signal domain, before we examined their combination by estimating a meta classifier. The two individual fNIRS classifiers were based on the evoked deflection from baseline HbO (HbO classifier) and HbR (HbR classifier). The EEG classifiers were based on induced band power changes (POW classifier) and the downsampled ERP waveform (ERP classifier).

#### 2.6.1. fNIRs features

Assuming an idealized haemodynamic stimulus response, i.e., a rise in HbO (HbO features) and a decrease in HbR (HbR features), stimulus-locked fNIRS features were extracted by taking the mean of the first few samples (i.e., *t_opt_* − $\frac{w}{2}$, …, *t_opt_*) substracted from the mean of the follwing samples (i.e., *t_opt_*, …, *t_opt_* + $\frac{w}{2}$) in all channels *c* of each trial, similar to Leamy et al. (2011). Equation 1 illustrates how the feature was calculated.

(1) $$\begin{array}{l} f_{c}^{HbO}=\frac{2}{w}\left(\sum_{t_{opt}}^{t_{opt}+\frac{w}{2}}\text{​}\text{​​​​}\Delta {\bar{\left[\text{HbO}\right]}}_{c}(t)-\text{​​}\sum_{t_{opt}-\frac{w}{2}}^{t_{opt}}\text{​}\text{​​​​​}\Delta {\bar{\left[\text{HbO}\right]}}_{c}(t)\right)\\ f_{c}^{HbR}=\frac{2}{w}\left(\sum_{t_{opt}}^{t_{opt}+\frac{w}{2}}\text{​}\text{​​​​}\Delta {\bar{\left[\text{HbR}\right]}}_{c}(t)-\text{​​}\sum_{t_{opt}-\frac{w}{2}}^{t_{opt}}\text{​}\text{​​​​​}\Delta {\bar{\left[\text{HbR}\right]}}_{c}(t)\right) \end{array}$$

#### 2.6.2. EEG features

For POW, the entire 10 s of all 10 channels were transformed to the spectral domain using Welch's method, and every other frequency component in the range of 3–40 Hz was concatenated to a 38-dimensional feature vector per channel. ERP features were always based on the first second (onset) of each trial. First, the ERP waveform underlied a median filter (*k_med_* = 5 ≈ 0.02s), followed by a moving average filter (*k_avg_* = 13 ≈ 0.05s). A final downsampling of the resulting waveform (*k_down_* = *k_avg_*) produced a 20-dimensional feature vector for each channel.

In the end, all features, i.e., HbO, HbR, POW, and ERP, were standardized to zero mean and unit standard deviation (z-normalization).

Four individual classifiers were trained based upon these four different feature types. Each classifier yielded a probability distribution across (the two) classes. Using those individual class probability values, we further evaluated a META classifier, based on decision fusion: The META classifier was based on the weighted sum *p*^meta^ = ∑_*m*_ *w*_*m*_ · *p_m_* of the class probability values *p_m_* of each of the four individual classifiers (*m* = HbO, HbR, POW, and ERP) with weight *w_m_*. The class with higher *p*^meta^, i.e., the maximum likelihood class, was then selected as the result of the META classifier.

The weights *w_m_* were estimated based on the classification accuracy on evaluation data (i.e., labeled data which is not part of the training data but available when building the classifier). Specifically, those classification accuracies that were higher than baseline (pure chance, i.e., 0.5 for the balanced binary classification conditions) were linearly scaled to the interval [0, 1], while those that were below baseline were weighted with 0, and thus, not incorporated. Afterwards, the weight vector *w* = [*w*_HbO_, *w*_HbR_, *w*_POW_, *w*_ERP_]^*T*^ was divided by its 1-norm in order to sum all of its elements to 1.

For the first three classifiers (HbO, HbR, and POW) a regularized linear discriminant analysis (LDA) classifier was employed (implemented following, Schlogl and Brunner, 2008 with a shrinkage factor of 0.5, as determined on evaluation data), while a soft-margin linear support vector machine (SVM) was used for the ERP classifier (using the LibSVM implementation by Chang and Lin, 2011 with default parameters). This was done because we expected the first three feature sets to be normally distributed (i.e., LDA is optimal), while we expected the more complex and variable temporal patterns of an ERP to require a more robust classification scheme. Note that this design choice was validated by evaluating both types of classifiers for all types of features on a representative subset of the data corpus. This ensured that in the reported results we used the classifier which leads to the optimal classification accuracy for every feature set.

For evaluation of the proposed hybrid BCI, we define a number of binary classification tasks. We call each different classification task a *condition*. Classification was performed for each modality and feature type separately as well as for the combined META classifier. In the subject-dependent case, we applied leave-one-trial-out cross-validation (resulting in 60 folds for 60 trials per subject). To estimate parameters of feature extraction and classification (*t_opt_* and *w* from Equation 1 for each fold, fusion weights *w_m_*), we performed another nested 10-fold cross-validation (i.e., in each fold, we have 53 trials for training and 6 trials (5 trials in the last fold) for evaluation) for the train set of each fold. The averaged accuracy in the inner cross-validation is used for parameter selection in the outer cross-validation. This procedure avoided overfitting of the parameters to the training data. In the subject-independent case, we performed leave-one-subject-out cross-validation, resulting in a training set of 660 trials and a test set of 60 trials per fold.

To evaluate those classifiers for the discrimination and detection of modality-specific processing, we define a number of binary classification conditions. Table 1 lists all defined classification conditions with the corresponding classes. All classification conditions are evaluated in a cross-validation scheme as described above. For each condition, we investigate both a subject-dependent classifier and a subject-independent classifier setup. As evaluation metric, we look at classification accuracy. Furthermore, we compare the performance of the individual classifiers (which only use one type of feature) with the META classifier and analyze the contribution of the two types of signals (EEG and fNIRS) to the different classification conditions. Additionally, we analyze the generalizability of the different detectors for modality-specific activity (lines 1–3 in Table 1) by evaluating the classifiers on trials with and without other independent perceptual and cognitive activity. Finally, we look at the classification performance on continuous data. For this purpose, we evaluate a subset of the classification conditions on windows extracted from continuous recordings without alignment to a stimulus onset.

**Table 1:** **Binary classification conditions for evaluation**.

**Condition**	**Class 1**	**Class 2**
AUD vs. VIS	AUD	VIS
AUD vs. IDLE	AUD	IDLE
VIS vs. IDLE	VIS	IDLE
allAUD vs. nonAUD	AUD, MIX	VIS, IDLE
allVIS vs. nonVIS	VIS, MIX	AUD, IDLE

*For each condition, we list the class labels which define the corresponding classes*.

## 3. Results

**Figure 7:**
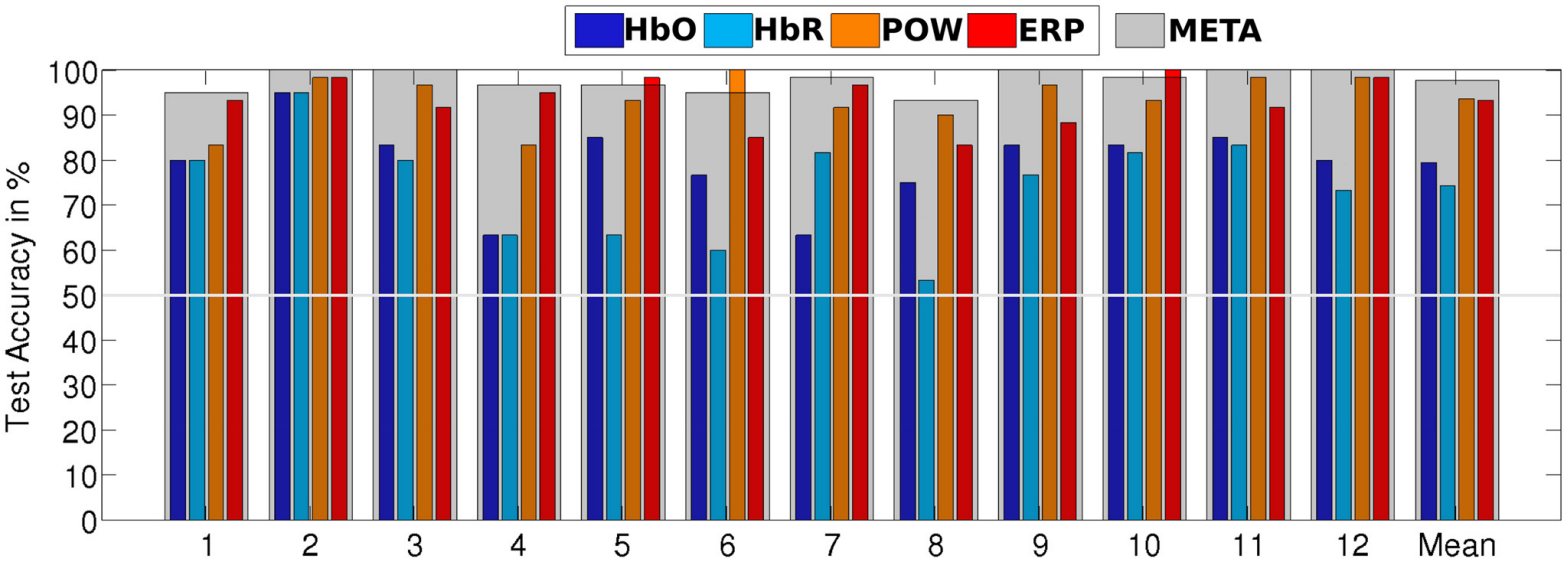
**Stimulus-locked recognition rates of AUD vs. VIS for subject-dependent, as well as for subject-independent classification**. Recognition rates of the META classifier are indicated by a gray overlay on top of the individual classifiers' bars.

Table 2 summarizes the recognition accuracy for all different conditions for the subject-dependent evaluation. The first entry is a discriminative task in which the classifier learns to separate visual and auditory perceptual activity. We see that for all four individual classifiers, a reliable classification is possible, albeit EEG-based features perform much better (HbO: 79.4% vs. POW: 93.6%). The fusion of all four classifiers, META, yields the best performance, significantly better (paired, one-sided *t*-test, α = 0.05 with Bonferroni-Holm correction for multiple comparisons) than the best individual classifier by a difference 4.2% absolute. This is in line with the results of the meta analysis by D'Mello and Kory (2012), who found modest, but consistent improvements by combining different modalities for the classification of inner states. Figure 7 shows a detailed breakdown of recognition results across all subjects for the example of AUD vs. VIS. We see that for every subject, recognition performance for every feature type was above the trivial classification accuracy of 50% and the performance of META was above 80% for all subjects.

**Table 2:** **Stimulus-locked classification accuracies (in %) for *subject-dependent* classification**.

	**HbO**	**HbR**	**POW**	**ERP**	**META**	***p***
AUD vs. VIS	79.4 (2.5)	74.3 (3.3)	93.6 (1.6)	93.3 (1.6)	97.8* (0.7)	0.006
AUD vs. IDLE	80.0 (2.7)	74.7 (3.1)	71.9 (3.0)	91.4 (1.7)	95.6* (1.6)	0.028
VIS vs. IDLE	83.8 (2.7)	78.1 (3.3)	90.7 (1.7)	81.9 (2.8)	96.4* (0.9)	0.002
allAUD vs. nonAUD	67.2 (3.1)	62.8 (3.3)	69.7 (2.0)	85.9 (1.7)	89.0* (1.5)	0.003
allVIS vs. nonVIS	68.5 (2.9)	64.7 (2.9)	91.5 (1.9)	81.9 (1.9)	94.8* (1.3)	0.019
Average	75.8	70.9	83.5	86.9	94.7	–

An asterisk in the META column indicates a significant improvement (α = 0.05) over the best corresponding individual feature type. Given in parantheses are standard errors of the mean. The last column indicates the *p* value of the statistical comparison of META and the best single-feature classifier. Highest classification accuracy for each condition is given in bold font.

In the next step, we evaluated subject-independent classification on the same conditions. The results are presented in Table 3. Averaged across all conditions, classification accuracy degrades by 6.5% compared to the subject-dependent results, resulting from higher variance caused by individual differences. Still, we managed to achieve robust results for all conditions, i.e., subject-independent discrimination visual and auditory processes is feasible. We therefore decided to report subsequent analyses for the subject-independent systems as those are much preferable from an HCI perspective.

**Table 3:** **Stimulus-locked classification accuracies (in %) for *subject-independent* classification**.

	**HbO**	**HbR**	**POW**	**ERP**	**META**	***p***
AUD vs. VIS	70.3 (2.2)	65.7 (2.2)	84.3 (2.2)	90.4 (1.3)	94.6* (1.3)	0.02
AUD vs. IDLE	64.0 (1.9)	61.9 (1.6)	66.1 (1.4)	84.2 (2.1)	86.9* (2.0)	0.002
VIS vs. IDLE	72.2 (2.8)	69.0 (4.0)	82.5 (2.9)	75.3 (2.6)	89.9* (1.8)	0.01
allAUD vs. nonAUD	60.6 (2.0)	58.8 (1.4)	41.7 (7.2)	**85.6** (2.1)	84.7 (1.3)	0.85
allVIS vs. nonVIS	62.7 (2.6)	62.0 (2.6)	84.2 (1.9)	73.1 (2.8)	86.7* (1.4)	0.003
Average	66.0	63.5	71.8	81.7	88.6	–

An asterisk in the META column indicates a significant improvement (α = 0.05) over the best corresponding individual feature type. Given in parantheses are standard errors of the mean. The last column indicates the *p* value of the statistical comparison of META and the best single-feature classifier. Highest classification accuracy for each condition is given in bold font.

The AUD vs. VIS condition denotes a discriminination task, i.e., it classifies a given stimulus as either auditory or visual. However, for an HCI application, those two processing modes are not mutually exclusive as auditory and visual perception can occur in parallel and can also be both absent in idle situations. We therefore need to define conditions which train a detector for specific perceptual activity, independently of the presence or absence of the other modality. Our first approach toward such a detector for auditory or visual perceptual activity is to define the AUD vs. IDLE and the VIS vs. IDLE conditions. A classifier trained on these conditions should be able to identify neural activity induced by the specific perceptual modality. In Tables 2, 3, we see that those conditions can be classified with high accuracy of 95.6% and 96.4% (subject-dependent), respectively. To test whether this neural activity can still be detected in the presence of other perceptual processes, we evaluate the classifiers trained on those conditions also on MIX trials. We would expect a perfect classifier to classify each of those MIX trials as VIS for the visual detector and AUD for the auditory detector. The top two rows of Table 4 summarize the results and show that the classifier still correctly detects the modality it is trained for in most cases.

**Table 4:** **Subject-independent classification accuracy of classifiers (in %) for AUD vs. IDLE and VIS vs. IDLE, evaluated on different trials from outside the respective training set**.

**Trained on…**	**Evaluated on…**	**HbO**	**HbR**	**POW**	**ERP**	**META**
AUD vs. IDLE	MIX	67.1	63.6	47.5	88.6	88.4
VIS vs. IDLE	MIX	69.3	68.4	69.0	84.7	77.6
AUD vs. IDLE	VIS	66.3	66.7	52.6	48.8	48.5
VIS vs. IDLE	AUD	59.5	61.4	49.3	50.5	48.2

A problem of those conditions is that it is not clear that a detector trained on them has actually detected specific visual or auditory activities. Instead, it may be the case that it has detected general cognitive activity which was present in both the AUD and VIS trials, but not in the IDLE trials. To analyze this possibility, we evaluated the classifier of the AUD vs. IDLE condition on VIS trials (and accordingly for VIS vs. IDLE evaluated on AUD). We present the results in the bottom two rows of Table 4. Both classifiers were very inconsistent in their results and “detected” modality-specific activity in nearly half of the trials, which actually did not contain such activity.

To train a classifier which is more sensitive for the modality-specific neural characteristics, we needed to include non-IDLE trials in the training data as negative examples. For this purpose, we defined the condition allAUD vs. nonAUD, where the allAUD class was defined as allAUD = {AUD, MIX} and the nonAD was defined as nonAUD = {IDLE, VIS}. Now, allAUD contains all data with auditory processing, while nonAUD contained all data without, but potentially with other perceptual activity. The condition allVIS vs. nonVIS was defined analogously. Tables 2, 3 document that a detector trained on these conditions was able to achieve a high classification accuracy. This result shows that the new detectors did not only learn to separate general activity from a resting state (as did the detectors defined earlier). If that would have been the case, we would have seen a classification accuracy of 75% or less: For example, if we make this assumption in the allVIS vs. nonVIS condition, we would expect 100% accuracy for the VIS, MIX and IDLE trials, and 0% accuracy for the AUD trials, which would be incorrectly classified as they contain general activity but none which is specific to visual processing. This baseline of 75% is outperformed by our classifiers for detection. This result indicates that we were indeed able to detect specific perceptual activity, even in the presence of other perceptual processes. For additional evidence, we look at how often the original labels (AUD, VIS, IDLE, MIX) were classified correctly in the two new detection setups by the META classifier. The results are summarized in Table 5 as a confusion matrix. We see that all classes are correctly classified in more than 75% of all cases, indicating that we detected the modality-specific characteristics in contrast to general cognitive activity.

**Table 5:** **Subject independent correct classification rate (in %) and confusion matrix for the allAUD vs. nonAUD and the allVIS vs. nonVIS conditions, broken down by original labels**.

	**AUD**	**VIS**	**IDLE**	**MIX**
allAUD	328	53	54	278
nonAUD	32	307	306	82
% correct	91.1	85.3	85.0	77.2
allVIS	65	339	64	318
nonVIS	295	21	296	42
% correct	81.9	84.2	82.2	88.3

**Figure 8:**
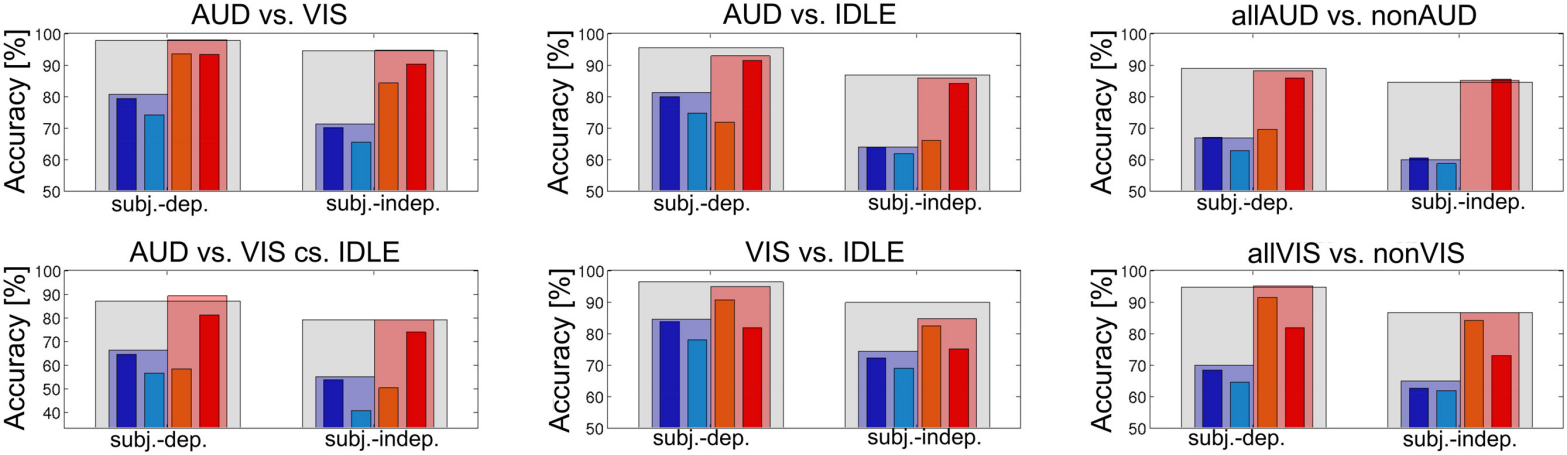
**fNIRS-META (red) vs. EEG-META (blue) evaluated for both subject-dependent and subject-independent classification for different conditions**.

The results we presented in Tables 2, 3 indicate that fusion was useful to achieve a high recognition accuracy. Still, there was a remarkable difference between the results achieved by the classifiers using fNIRS features and by classifiers using EEG features. This was true across all investigated conditions and for both subject dependent and subject independent classification. We suspect that the advantage of the META classifier was mostly due to the combination of the two EEG based classifiers. In Figure 8, we investigated this question by comparing two fusion classifiers EEG-META and fNIRS-META which combined only the two fNIRS features or the two EEG features, respectively. The results show that for the majority of the conditions, the EEG-META classifier performed as good as or even better than the overall META classifier. However, the fNIRS features contributed significantly to the classification accuracy for both conditions AUD vs. IDLE and VIS vs. IDLE (*p* = 0.003 and *p* = 0.01, respectively for the difference of EEG-META and META in the subject-dependent case).

To exclude that the difference was due to the specific fNIRS feature under-performing in this evaluation, we repeated the analysis with other established fNIRS features (average amplitude, value of largest amplitude increase or decrease). The analysis showed that we could not achieve improvements by exchanging fNIRS feature calculation compared to the original feature. We conclude that the difference in accuracy was not caused by decisions during feature extraction. Overall, we see that fNIRS-based features were outperformed by the combination of EEG based features for the most investigated conditions but that it could still contribute to a high classification accuracy in some of the cases.

**Figure 9:**
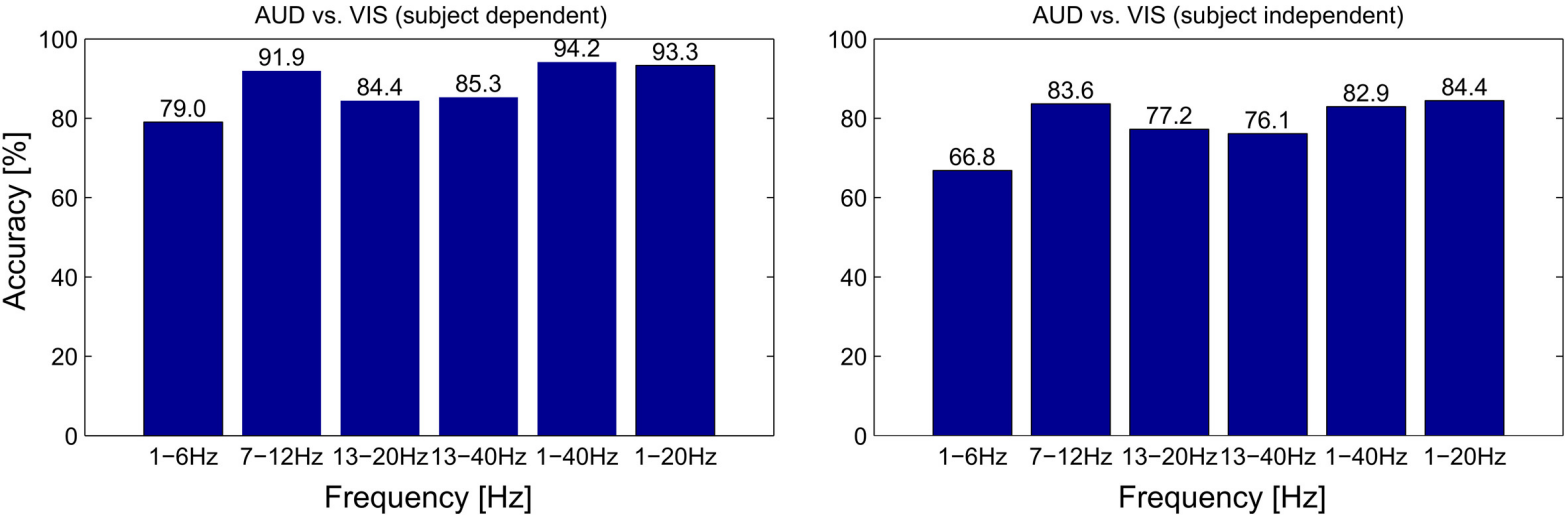
**Classification accuracy for different filter boundaries for the POW feature set, evaluated for both subject-dependent (left half) and subject-independent (right half) classification for different conditions**.

There are however some caveats to the dominance of EEG features. First, the ERP classifier is the only one of the four feature types which is fundamentally dependent on temporal alignment to the stimulus onset and therefore not suited for many applications of continuous classification. While the employed fNIRS features also use information on the stimulus onset (as they essentially characterize the slope of the signal), only the ERP features rely on specific oscillatory properties in a range of milliseconds (compare Figures 5 and 4), which cannot be extracted reliably without a stimulus locking. Second, concerning the POW classifier, we see in Figure 6 a large difference in alpha power between VIS and AUD. As both types of trials induce cognitive activity, we did not expect the AUD trials to exhibit alpha power (i.e., idling rhythm) nearly at an IDLE level. We cannot completely rule out that this effect is caused at least in parts by the experimental design (e.g., because visual stimuli and auditory stimuli differed in complexity) or subject selection (e.g., all subjects were familiar with similar recording setups and therefore easily relaxed). Therefore, we need to verify that the discrimination ability of the POW classifier does not solely depend on differences in alpha power. For that purpose, we repeated the evaluation of AUD vs. VIS with different sets of band pass filters, of which some excluded the alpha band completely. Results are summarized in Figure 9. We see that as expected, feature sets including the alpha band performed best. Accuracy dropped by a maximum of 9.4% relative when removing the alpha band (for the subject dependent evaluation from 1–40 Hz to 13–40 Hz). This indicates the upper frequency bands still contain useful discriminating information.

The previous analysis showed that different features contributed to different degrees to the classification result. Therefore, we were interested in studying which features were stable predictors of the ground truth labels on a single trial basis. The successful person-independent classification was already an indication that such stable, generalizable features exist. To investigate which features contributed to the detection of different modalities, we calculated the correlation of each feature with the ground truth labels for the conditions VIS vs. IDLE and AUD vs. IDLE.

For the POW features, we ranked the electrode by their highest absolute correlation across the whole frequency range for each subject. To see which features predicted the ground truth well across all subjects, we averaged those ranks. The resulting average rankings are presented in the first two columns of Table 6. We note that for the VIS vs. IDLE condition, electodes at the occipital cortex were most strongly correlated to the ground truth. In contrast, for the AUD vs. IDLE condition, those electrodes can be found at the bottom of the ranking. For this condition, the highest ranking electrodes were at the central-midline (it was expected that electrodes above the auditory cortex would not contribute strongly to the AUD vs. IDLE condition as activity in the auditory cortex cannot be captured well by EEG). The low *SD* also indicates that the derived rankings are stable across subjects. We can therefore conclude that the POW features were generalizable and neurologically plausible.

**Table 6:** **Average rankings of electrode positions derived from correlation of POW and ERP features to ground truth labels**.

**Rank**	**VIS vs. IDLE**	**AUD vs. IDLE**	**VIS vs. IDLE**	**AUD vs. IDLE**
1	Oz (2.5)	Pz (2.3)	O1 (2.6)	Cz (3.0)
2	O2 (2.2)	Cz (2.4)	O2 (2.9)	Fz (1.4)
3	Pz (2.2)	Fz (1.7)	Oz (3.1)	Pz (3.0)
4	TP8 (3.2)	TP8 (2.3)	TP8 (3.0)	TP7 (2.7)
5	TP7 (2.8)	TP7 (1.9)	Fz (2.2)	FT8 (2.7)
6	Fz (3.4)	FT7 (3.0)	TP7 (2.9)	TP8 (2.3)
7	O1 (1.5)	O2 (2.8)	Pz (3.1)	FT7 (2.4)
8	Cz (2.2)	FT8 (3.0)	Cz (2.3)	O1 (0.8)
9	FT8 (3.6)	O1 (3.3)	FT7 (2.6)	Oz (1.6)
10	FT7 (2.4)	Oz (2.6)	FT8 (3.0)	O2 (2.1)

We then ranked the frequency band features by their highest absolute correlation across the whole electrode set for each subject and average those ranks across subjects. We observed the highest average ranks at 9.5 Hz and at 18.5 Hz. Especially for the first peak in the alpha band, we observed a low *SD* of 6.2, which indicates that those features were stable across subjects.

For the ERP features, we repeated this analysis (with time windows in place of frequency bands). The two rightmost columns of Table 6 show a similar picture as for the POW features regarding the contribution of individual electrodes: Features from electrodes at the occipital cortex were highly discriminative in the VIS vs. IDLE condition, features from central-midline electrodes carried most information in the AUD vs. IDLE condition. Regarding time windows, we observe the best rank for the window starting at 312ms, which corresponds well to the expected P300 component following a stimulus onset. With a *SD* of 2.9, this feature was also ranked highly across all subjects.

To investigate the reliability of the derived rankings, we conducted Friedman tests on the rankings of all participants. Those showed that all investigated rankings (with one exception) yielded a significant difference in average ranks of the items. The resulting *p*-values are given in Table 7. This indicates that the rankings actually represent a reliable, person-independent ordering of features.

**Table 7:** **Resulting *p*-values for Friedman tests to investigate whether the calculated average feature rankings are statistically significant**.

**Feature**	**Condition**	**Ranking by …**	***p*-value**
ERP	AUD vs. IDLE	Electrodes	<10^−5^
ERP	AUD vs. IDLE	Time windows	<10^−10^
ERP	VIS vs. IDLE	Electrodes	0.12
ERP	VIS vs. IDLE	Time windows	<10^−10^
POW	AUD vs. IDLE	Electrodes	<10^−3^
POW	AUD vs. IDLE	Frequency bands	<10^−10^
POW	VIS vs. IDLE	Electrodes	<10^−2^
POW	VIS vs. IDLE	Frequency bands	<10^−10^

The analysis for fNIRS features differed from the EEG feature analysis because of the signal characteristics. For example, the fNIRS channels were spatially very close to each other and highly correlated. Therefore, we did not look at features from single fNIRS channels. Instead, we differentiated between the different probes. For the VIS vs. IDLE condition, the channel which yielded the highest absolute correlation was located above the visual cortex for 75% of all subjects (averaged across both hBO and HbR). For the AUD vs. IDLE condition, the channel with the highest absolute correlation was located above the auditory cortex for 91.6% of all subjects. This indicates that the fNIRS signals also yielded neurologically plausible features which generalized well across subjects. When comparing HbO and HbR features, the HbO features were correlated slightly higher to the ground truth (19.6% higher maximum correlation) than the HbR features, which corresponds to their higher classification accuracy.

The classification setups which we investigated up to this point are all defined on trials which are locked at the onset of a stimulus. The detection of onsets of perceptual activity is an important use case for HCI applications: The onset of a perceptual activity often marks a natural transition point to react to a change of user state. On the other hand, there are use cases where the detection of ongoing perceptual activity is relevant. To investigate how the implemented classifiers perform on continuous stimulus presentation, we evaluated classification and detection on the three continuous segments (60s of each AUD, VIS, MIX) which were recorded in the first block for each subject. As data is sparse for those segments, we only regard the subject-independent approach. To extract trials, the data was segmented into windows of a certain length (overlapping by 50%). We evaluated the impact of the window size on the classification accuracy: For window sizes of 1, 2, 4, 8, and 16 s, we end up with 120, 60, 30, 15, and 8 windows per subject and class, respectively. Those trials are not aligned to a stimulus onset. We used the same procedure to extract POW features as for the onset-locked case. The ERP feature was the basis of the best non-fusion classifier but is limited to detecting stimulus onsets. Therefore, we excluded it from the analysis to investigate the performance of the remaining classifiers. For both feature types based on fNIRS, we modified the feature extraction to calculate the mean of the window, normalized by the mean of the already elapsed data. The other aspects of the classifier were left unchanged.

**Figure 10:**
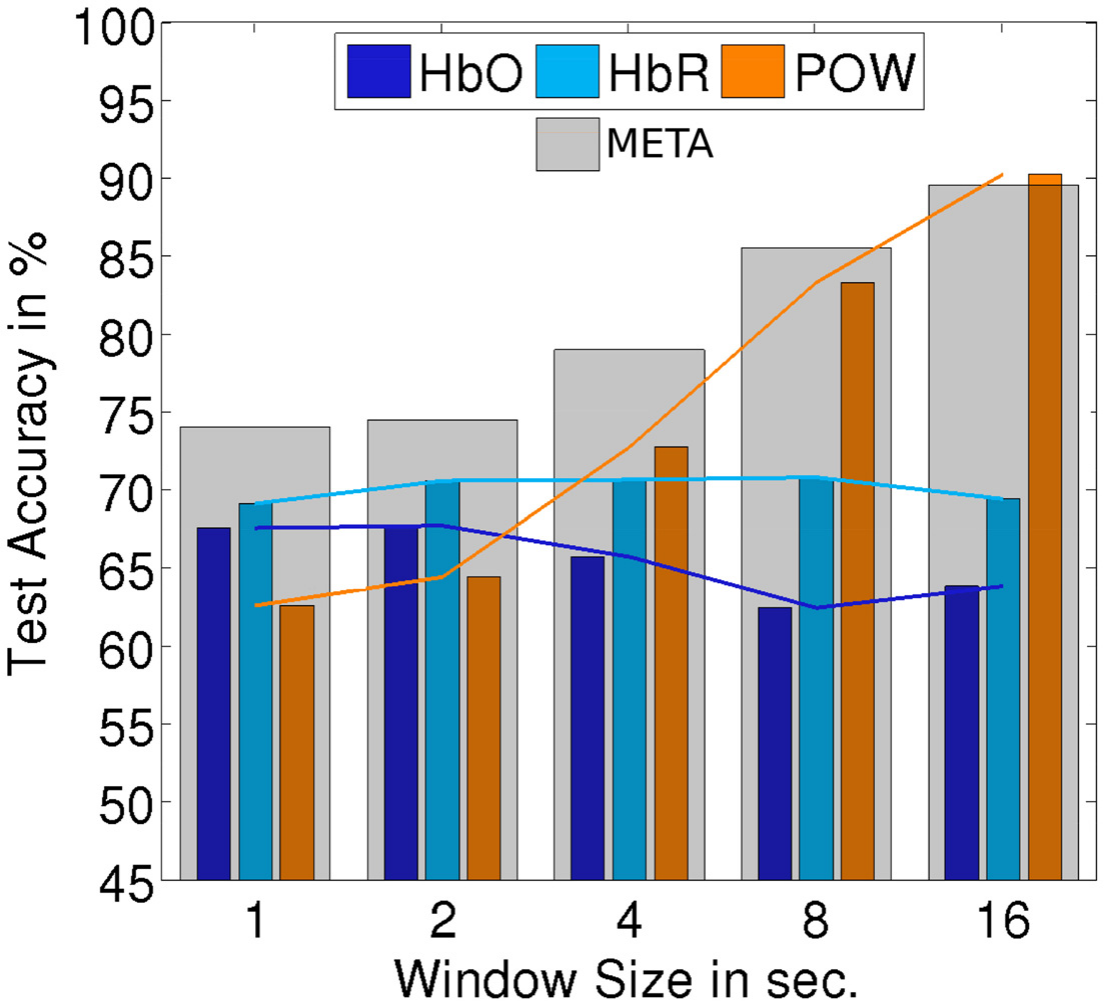
**Accuracy for subject-independent classification of AUD vs. VIS on continuous data**. Results are in dependency of window size.

**Figure 11:**
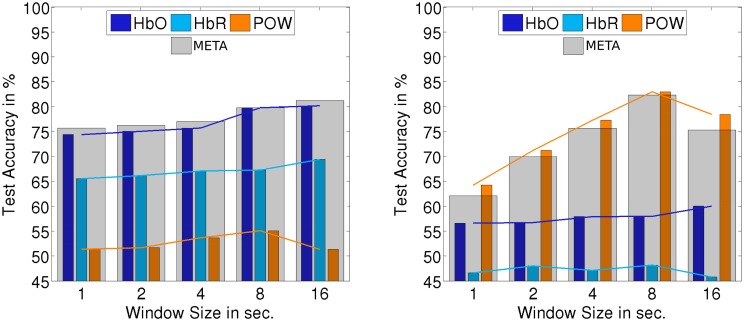
**Accuracy for subject-independent classification of allAUD vs. nonAUD (left) and allVIS vs. nonVIS (right) on continuous data**. Results are in dependency of window size.

Figures 10, 11 summarize the results of continuous evaluation. The results are mostly consistent with our expectations and the previous results on stimulus-locked data. For all three regarded classification conditions, we achieve an accuracy of more than 75% for META, i.e., reliable classification does not solely depend on low-level bottom-up processes at the stimulus onset. Up to the threshold of 16 s, there was a benefit of using larger windows for feature calculation. Note that with growing window size, the number of trials for classification drops, which also has an impact on the confidence interval for the random baseline (Mueller-Putz et al., 2008). The upper limit of the 1% confidence interval is 52.4% for a window size of 1 s, 53.4% for 2 s, 54.9% for 4 s, 56.9% for 8 s, and 59.5% for 16 s. This should be kept in mind when interpreting the results, especially for larger window sizes. The EEG feature yields a better classification accuracy than the two fNIRS-based classifiers in two of the three cases. For the allAUD vs. nonAUD situation however, the POW classifier does not exceed the random baseline and only the two fNIRS based classifiers can achieve satisfactory results. Therefore, we see that when ERP features are missing in the continuous case, the fNIRS features can substantially contribute to classification accuracy in the case of allAUD vs. nonAUD.

## 4. Discussion

The results from the previous section indicate that both the discrimination and detection of modality-specific perceptual processes in the brain is feasible both in a subject-dependent as well as a subject-independent setup with high recognition accuracy. We see that the fusion of multiple features from different signal types led to improvement in recognition accuracy significantly. However, in general fNIRS-based features were outperformed by features based on the EEG signal. In the future, we will look closer into other reasons for this gap and potential remedies for it. One difference between fNIRS and EEG signals is the lack of advanced artifact removal techniques for fNIRS that have been applied with some success in other research on fNIRS BCIs (Molavi and Dumont, 2012). Another difference is that the coverage of fNIRS optodes was limited mainly to the sensory areas, but our EEG measures may include robust effects generated from other brain regions, such as the frontal-parietal network. Activities in these regions may be reflecting higher cognitive processes triggered by the different modalities, other than purely perceptual ones. It may be worthwhile to extend the fNIRS setup to include those regions as well. Still, we already saw that fNIRS features can contribute significantly to certain classification tasks. While evaluation on stimulus-locked data allows a very controlled evaluation process and is supported by the very high accuracy we can achieve, this condition is not very realistic for most HCI applications. In many cases, stimuli will continue over longer periods of time. Features like the ERP feature explicitly model the onset of a perceptual process but will not provide useful information for ongoing processes. In future work, we will investigate such continuous classification on the longer, continuous data segments of the recorded corpus.

Following the general guidelines of Fairclough (2009), one limitation in validity of the present study is the fact that there may be other confounding variables that can explain the differences in the observed neurological responses to the stimuli of different modalities. Subjects were following the same task for all types of stimuli; still, factors like different memory load or increased need for attention management due to multiple parallel stimuli for visual trials may contribute to the separability of the classes. We address this partially by identifying the expected effects, for example in Figure 4 comparing fNIRS signals from visual and auditory cortex. Also the fact that detection of both visual and auditory processing worked on MIX trials shows that the learned patterns were not only present in the dedicated data segments but were to some extend generalizable. Still, we require additional experiments with different tasks and other conditions to reveal whether it is possible to train a fully generalizable detector and discriminator for perceptual processes. Finally, we also have to look into a more granular model with a higher sensitivity than the presented dichotomic characterization of perceptual workload.

The evaluation was performed in a laboratory setting but with natural and complex stimulus material. The results indicate that such a system is robust enough to use it for the improvement an HCI system in a realistic scenario. We saw that both EEG and fNIRS contributed to a high classification accuracy; in most cases, the results for the EEG-based classifiers were more accurate than for the fNIRS based ones. Whether the additional effort which is required to apply and evaluate a hybrid BCI (compared to a BCI with only one signal type) depends on the specific application. When only one specific classification condition is relevant (e.g., to detect processing of visual stimuli), there is always a single optimal signal type which is sufficient to achieve robust classification. The benefit of a hybrid system is that it can potentially cover multiple different situations for which no generally superior signal type exists. Another aspect for the applicability of the presented system for BCI is the response latency, which also depends on the choice of employed features. The ERP features react very rapidly to but are limited to situations, in which a stimulus onset is present. Such short response latency (less than 1 s) may be useful when an HCI system needs to immediately switch communication channels or interrupt communication to avoid perceptual overload of the user (for example, when the user unexpectedly engages in a secondary task besides communicating with the HCI system). In such situations, the limitation to onsets is also not problematic. On the other hand, if the system needs to assume that the user is already engaged in a secondary task when it starts to observe him or her (i.e., to determine the initial communication channel at the beginning of a session), it is not sufficient anymore to only respond to stimulus onsets. For those cases, it may be worthwhile to accept the latency required by the fNIRS features and also the POW feature for a classification of continuous perceptual activity.

We conclude that we demonstrated the first passive hybrid BCI for the discrimination and detection of perceptual activity. We showed that robust classification is possible both in a subject-dependent and a subject-independent fashion. While the EEG features outperformed the fNIRS features for most parts of the evaluation, the fusion of multiple signals and features was beneficial and increased the versatility of the BCI.

### Conflict of interest statement

The authors declare that the research was conducted in the absence of any commercial or financial relationships that could be construed as a potential conflict of interest.

## Footnotes

^1^compiled by Scott Prahl using data from: W. B. Gratzer, Med. Res. Council Labs, Holly Hill, London, and N. Kollias, Wellman Laboratories, Harvard Medical School, Boston.
